# A lung rescue team improves survival in obesity with acute respiratory distress syndrome

**DOI:** 10.1186/s13054-019-2709-x

**Published:** 2020-01-15

**Authors:** Gaetano Florio, Matteo Ferrari, Edward A. Bittner, Roberta De Santis Santiago, Massimiliano Pirrone, Jacopo Fumagalli, Maddalena Teggia Droghi, Cristina Mietto, Riccardo Pinciroli, Sheri Berg, Aranya Bagchi, Kenneth Shelton, Alexander Kuo, Yvonne Lai, Abraham Sonny, Peggy Lai, Kathryn Hibbert, Jean Kwo, Richard M. Pino, Jeanine Wiener-Kronish, Marcelo B. P. Amato, Pankaj Arora, Robert M. Kacmarek, Lorenzo Berra, For the investigators of the lung rescue team, Gaetano Florio, Matteo Ferrari, Edward A. Bittner, Roberta De Santis Santiago, Massimiliano Pirrone, Jacopo Fumagalli, Maddalena Teggia Droghi, Cristina Mietto, Riccardo Pinciroli, Sheri Berg, Aranya Bagchi, Kenneth Shelton, Alexander Kuo, Yvonne Lai, Abraham Sonny, Peggy Lai, Kathryn Hibbert, Jean Kwo, Richard M. Pino, Jeanine Wiener-Kronish, Marcelo B. P. Amato, Pankaj Arora, Robert M. Kacmarek, Lorenzo Berra, David Imber, Daniel Fisher, Daniel Chipman, Carolyn LaVita

**Affiliations:** 1 Department of Anesthesia, Critical Care and Pain Medicine, Massachusetts General Hospital, Harvard Medical School, 55 Fruit Street, Boston, MA 02141 USA; 2 Division of Pulmonary and Critical Care, Massachusetts General Hospital, Boston, MA USA; 3 Pulmonary Division, Cardio-Pulmonary Department, Heart Institute (Incor), Hospital Das Clinicas da FMUSP, University of Sao Paulo, Sao Paulo, Brazil; 4 Division of Cardiovascular Disease, University of Alabama at Birmingham, Birmingham, AL USA; 5 Department of Respiratory Care, Massachusetts General Hospital and Harvard Medical School, Boston, MA USA

**Keywords:** ARDS, Obesity, Mechanical ventilation, Cardiopulmonary physiology, Mortality

## Abstract

**Background:**

Limited data exist regarding ventilation in patients with class III obesity [body mass index (BMI) > 40 kg/m^2^] and acute respiratory distress syndrome (ARDS). The aim of the present study was to determine whether an individualized titration of mechanical ventilation according to cardiopulmonary physiology reduces the mortality in patients with class III obesity and ARDS.

**Methods:**

In this retrospective study, we enrolled adults admitted to the ICU from 2012 to 2017 who had class III obesity and ARDS and received mechanical ventilation for > 48 h. Enrolled patients were divided in two cohorts: one cohort (2012–2014) had ventilator settings determined by the ARDSnet table for lower positive end-expiratory pressure/higher inspiratory fraction of oxygen (*standard protocol-based cohort*); the other cohort (2015–2017) had ventilator settings determined by an individualized protocol established by a lung rescue team (*lung rescue team cohort*). The lung rescue team used lung recruitment maneuvers, esophageal manometry, and hemodynamic monitoring.

**Results:**

The standard protocol-based cohort included 70 patients (BMI = 49 ± 9 kg/m^2^), and the lung rescue team cohort included 50 patients (BMI = 54 ± 13 kg/m^2^). Patients in the standard protocol-based cohort compared to lung rescue team cohort had almost double the risk of dying at 28 days [31% versus 16%, *P* = 0.012; hazard ratio (HR) 0.32; 95% confidence interval (CI95%) 0.13–0.78] and 3 months (41% versus 22%, *P* = 0.006; HR 0.35; CI95% 0.16–0.74), and this effect persisted at 6 months and 1 year (incidence of death unchanged 41% versus 22%, *P* = 0.006; HR 0.35; CI95% 0.16–0.74).

**Conclusion:**

Individualized titration of mechanical ventilation by a lung rescue team was associated with decreased mortality compared to use of an ARDSnet table.

**Electronic supplementary material:**

The online version of this article (10.1186/s13054-019-2709-x) contains supplementary material, which is available to authorized users.

## Background

Approximately 40% of all adults in the USA are obese [1]. The prevalence of the most severe form of obesity [class III obesity: body mass index (BMI) > 40 kg/m^2^] is approaching 10% (> 30 million Americans) [2]. Little has been done in the intensive care unit (ICU) to study this healthcare epidemic, which is associated with overall reduced life expectancy [3]. A common cause of ICU admission for patients with class III obesity is acute respiratory distress syndrome (ARDS) [4], often leading to dependency on mechanical ventilation, high incidence of tracheostomy [5], severe kidney failure [6], multiple organ failure, and significantly higher all-cause mortality [7, 8].

Appropriate protective mechanical ventilation is the cornerstone for treatment of patients with ARDS [9–13]. To improve lung healing and survival, several ventilation strategies have been tested with different degrees of success. Positive end-expiratory pressure (PEEP) and lung recruitment maneuvers (LRM) are two ventilation strategies aimed to decrease overstretching of lung parenchyma and cyclic opening and closing of small airways and alveoli (i.e., barotrauma, volutrauma, and atelectrauma). Obesity has been an exclusion criterion in most of the major ARDS trials testing different modalities to titrate mechanical ventilation [9, 11, 14, 15]. Despite a lack of evidence of benefit to patients with obesity, most clinicians use the ARDSnet PEEP/inspiratory fraction of oxygen (F_i_O_2_) protocol [9, 11] to titrate mechanical ventilation in all ARDS patients.

Our recent studies confirm that pleural pressure in patients with class III obesity is higher than in patients with lean body habitus [16–19]. Increased pleural pressure significantly reduces lung volume (especially functional residual capacity) and leads to formation of atelectasis, which is associated with shunting and hypoxemia [17–19]. Patients with obesity often have highly recruitable lungs, and common PEEP levels used in the ICU are not sufficient to prevent atelectasis [17–19]. As a result, in this population, only an individualized physiological titration of PEEP with a LRM is effective to counter the detrimental effects of increased pleural pressure, resulting in lung re-expansion [17–19].

We tested the hypothesis that implementation of a specialized team (the lung rescue team) exclusively assessing patients with both obesity and ARDS would reduce mortality due to individualized physiologic treatment of such unique patients. To provide individualized titration of mechanical ventilation in patients with class III obesity, the Massachusetts General Hospital (MGH) Respiratory Care Service together with the Critical Care Group implemented a lung rescue team in 2014.

## Methods

### Patients and measurements

The Institutional Review Board approved the study (MGH-IRB-2017P000544) with waiver of patient consent.

Adult patients (≥ 18 years of age) with the following entry criteria were enrolled: (I) class III obesity (BMI > 40 kg/m^2^), (II) diagnosis of ARDS [4], (III) mechanical ventilation for > 48 h, and (IV) admission to MGH surgical or medical ICU from January 1, 2012, to December 31, 2017. None of these patients participated in any MGH ARDS ongoing interventional trials.

This retrospective study compared two cohorts of patients. During 2012–2014, mechanical ventilation settings of the first cohort of patients (standard protocol-based cohort) was titrated according to ARDSnet PEEP/F_i_O_2_ guidelines [9, 11]. During 2015–2017, mechanical ventilation of the second cohort of patients (lung rescue team cohort) was treated by the lung rescue team.

During ICU admission and the first 4 days of mechanical ventilation, patient characteristics together with cardiopulmonary and hemodynamic were recorded. Patients’ outcomes up to 1-year follow-up were also recorded.

### Interventions

All ARDS patients at MGH were ventilated in volume-controlled or pressure-controlled ventilation mode, with tidal volumes of 4–8 mL/kg of predicted body weight while maintaining plateau pressure of < 28 cmH_2_O and respiratory rate titrated to maintain 88%–95% SpO_2_ and permissive hypercapnia with pH > 7.25 and PaCO_2_ < 60 mmHg [20, 21]. To prevent ventilation asynchrony and for ARDS treatment, patients were paralyzed with cisatracurium to suppress train-of-four to 0 to 1 [22].

**Fig. 1:**
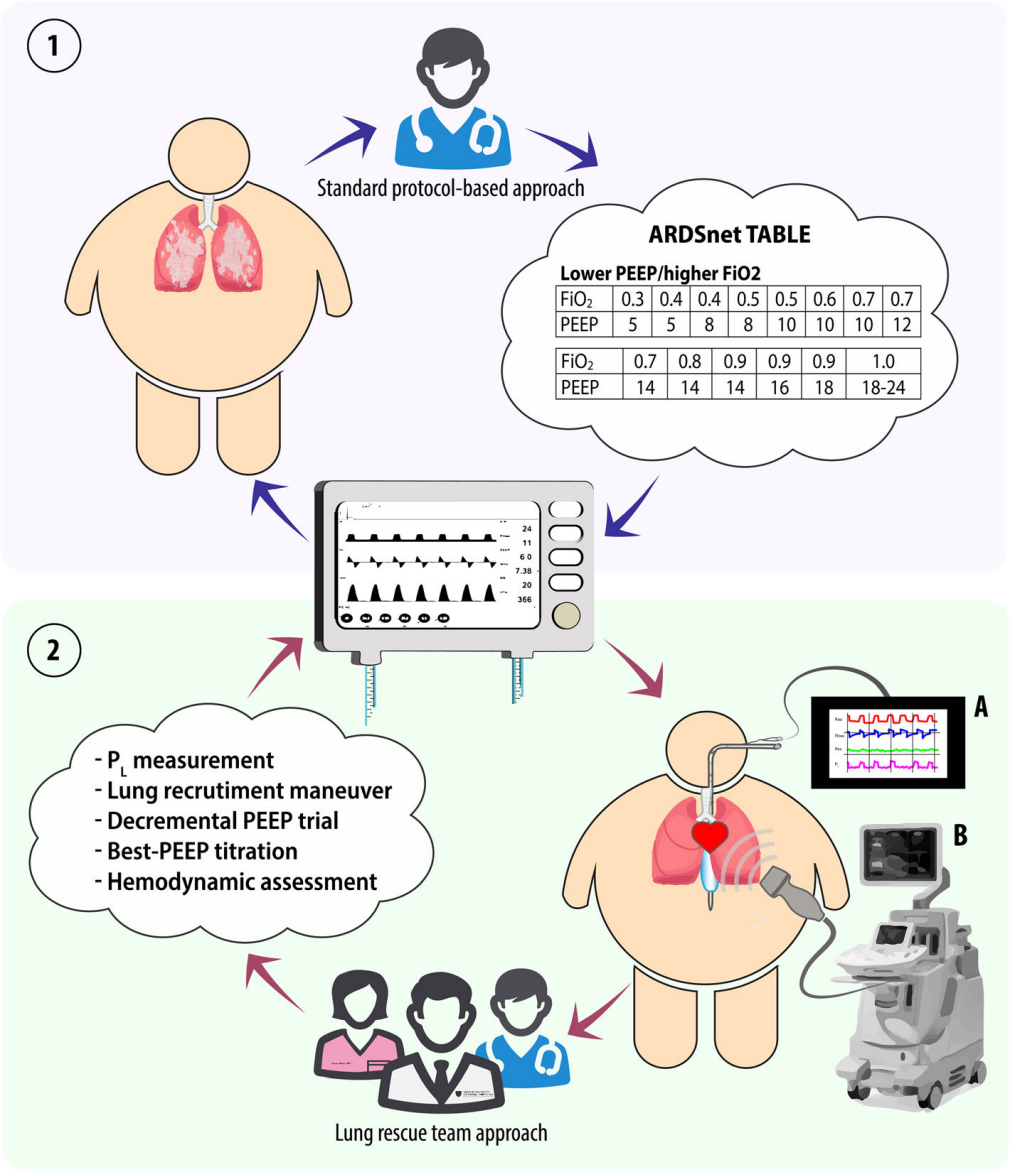
Standard protocol-based approach versus lung rescue team approach. According to the standard protocol-based approach, ARDS patients were essentially treated setting the mechanical ventilator in accordance with the indications provided by ARDSnet tables (panel **1**). Conversely, an individualized lung rescue team approach (panel **2**) involved a thorough (multidisciplinary) assessment of respiratory mechanics, including esophageal pressure monitoring (**2**, A), as well as the patient’s response to lung recruitment. The best-PEEP was titrated based on a decremental PEEP trial, while hemodynamics was assessed by means of transthoracic echocardiography (**2**, B). PEEP, positive end-expiratory pressure; F_i_O_2_, inspiratory fraction of oxygen; P_L_, transpulmonary pressure

For the standard protocol-based cohort (Fig. 1, panel 1), ventilation of patients with class III obesity was managed using ARDSnet PEEP/F_i_O_2_ tables [11]. Due to the absence of benefit associated with the higher PEEP/lower F_i_O_2_ table [11], the lower PEEP/higher F_i_O_2_ table was used.

For the lung rescue team cohort (Fig. 1, panel 2), ventilation was started based on the lower PEEP/higher F_i_O_2_ table. Subsequently, ventilation was titrated by the lung rescue team, composed of a critical care physician, two critical care fellows, and an ICU respiratory therapist. The lung rescue team represents the clinical evolution of the research activity performed by our group. In recent years, our group extensively investigated ventilatory management of patients with obesity and acute respiratory failure, and these studies often led to a dramatic improvement in the levels of hypoxemia. Consequently, the ICUs of MGH started to request a respiratory consult and a clinical team was implemented. The lung rescue team evaluated patients within the first day from the beginning of mechanical ventilation. The clinical decisions of the lung rescue team were based on multiple measurements of respiratory mechanics and hemodynamics; mechanical ventilation was accurately titrated using LRM and choosing the correct value of PEEP matching information from decremental PEEP trial, end-expiratory transpulmonary pressure measurements, and the use of electrical impedance tomography. Hemodynamics was carefully analyzed through standard hemodynamic parameters and right heart echocardiography with the aim to intensively study the interaction between lung and heart/vascular function [23].

In both cohorts of patients, the weaning process from mechanical ventilation was performed according to the recommendations of the 2005 International Consensus Conference [24].

### Statistical analysis

Baseline characteristics, respiratory mechanics, and hemodynamics during the first 4 days of ICU admission and outcomes were compared between the two groups with two-sample parametric or nonparametric tests as appropriate. Normality of distribution was assessed using the Shapiro-Wilk test. T-test/Wilcoxon rank-sum and chi-square test were used for group comparison among continuous/categorical and categorical/categorical variables, respectively.

The primary outcome was mortality at 28 days and was decided a priori during the study design process. The effect of lung rescue ventilation strategy on mortality up to 1-year follow-up was assessed using Kaplan-Meier curves, and hazard ratio was calculated using the Cox proportional hazard model. Kaplan-Meier analysis was used to examine unadjusted differences in survival in the two groups. The Cox proportional hazard model was used to examine differences in survival after adjusting for predetermined potential confounders (BMI, age, APACHE, PaO2/FiO2 ratio). A two-sided *P* value of < 0.05 was considered statistically significant. Data from all patients admitted to the ICU that met inclusion criteria were collected and included in the analysis; an a priori sample size calculation was not performed. All statistical analysis and all graphs were performed using STATA version 13 (STATA Corp., USA).

Please see supplementary materials for details regarding patient screening, recordings, measurements, and interventions.

## Results

### Patient characteristics

From 2012 to 2014, 70 ARDS patients (BMI = 49 ± 9 kg/m^2^) were managed according to a standard protocol-based approach. From 2015 to 2017, 50 ARDS patients (BMI = 54 ± 13 kg/m^2^) were managed by the lung rescue team. Upon ICU admission, patients in the first cohort were slightly older (57 ± 13 years versus 52 ± 14 years, *P* = 0.03). No other baseline comorbidities differed between the groups (Table 1).

**Table 1:** Baseline characteristics of patients

	Standard protocol-based cohort	Lung rescue team cohort	*P*
Patients, *n* (%)	70 (100)	50 (100)	
Women, *n* (%)	37 (53)	23 (46)	0.46
Caucasian, *n* (%)	64 (91)	48 (96)	0.32
Others, *n* (%)	6 (9)	2 (4)	
Age, years, mean (SD)	57 (13)	52 (14)	0.03
BMI, kg/m^2^ , mean (SD)	49 (9)	54 (13)	0.11
Cause of admission, *n* (%)			
Postoperative respiratory failure after elective surgery	14 (20)	11 (22)	0.79
Postoperative respiratory failure after urgent surgery	17 (24)	11 (22)	0.77
Medical	39 (56)	28 (56)	0.85
Pneumonia	12 (17)	9 (18)	0.96
Septic shock	14 (20)	12 (24)	0.62
Others	13 (18)	7 (14)	0.56
APACHE II, mean (SD)	19 (7)	19 (8)	0.82
SOFA, mean (SD)	9.9 (3.6)	9.8 (3.5)	0.62
Comorbidities, *n* (%)			
Diabetes	29 (41)	21 (42)	0.95
Oral agents	14 (20)	10 (20)	0.96
Oral agents + insulin	15 (21)	11 (22)	0.96
Hypertension	48 (68)	34 (68)	0.95
Asthma	9 (13)	5 (12)	0.89
COPD	20 (28)	14 (28)	0.94
OSA	18 (25)	14 (28)	0.78
Smoking	24 (34)	21 (42)	0.39
Actual	12 (17)	9 (18)	0.90
Former	12 (17)	12 (24)	0.35
CHF	15 (21)	9 (18)	0.64
Stroke, TIA	4 (6)	2 (4)	0.67
CKD	11 (16)	6 (12)	0.56
PVD	7 (10)	6 (12)	0.73
AF	12 (17)	9 (18)	0.90
Cancer	5 (7)	7 (14)	0.22

*Abbreviation*: *SD* standard deviation, *BMI* body mass index, *APACHE* acute physiologic assessment and chronic health evaluation scoring, *SOFA* sequential organ failure assessment, *COPD* chronic obstructive pulmonary disease, *OSA* obstructive sleep apnea, *CHF* congestive heart failure, *TIA* transient ischemic attack, *CKD* chronic kidney disease, *PVD* peripheral vascular disease, *AF* atrial fibrillation (chronic atrial fibrillation on anticoagulant therapy)

### Ventilation settings

At ICU admission, P_a_O_2_/F_i_O_2_ was higher in the standard protocol-based cohort (PaO_2_/FiO_2_ ratio of 197 mmHg [CI95% 177–217], compared to 154 mmHg [CI95% 127–179] in the lung rescue group, *P* = 0.003) (Table 2 and Additional file 1: Table S1). No other differences were observed in baseline lung mechanics between the two cohorts (Table 2 and Additional file 1: Table S1).

**Table 2:** Ventilation settings and hemodynamics—standard protocol-based cohort and lung rescue team cohort

Variable	Group	Day 1		Day 2		Day 3		Day 4	
PEEP, cmH_2_O , mean (CI 95%)	Standard protocol-based cohort	9 (8–10)	*P* = 0.20	9 (8–10)	*P* < 0.001	9 (8–10)	*P* < 0.001	9 (8–10)	*P* < 0.001
	Lung rescue team cohort	9 (9–10)		19 (18–20)		20 (18*–*21)		20 (18–21)	
TV/IBW, mL/kg , mean (CI 95%)	Standard protocol-based cohort	6.4 (6.2–6.6)	*P* = 0.33	6.5 (6.3–6.8)	*P* = 0.10	6.4 (6.1*–*6.6)	*P* = 0.21	6.5 (6.3–6.7)	*P* = 0.11
	Lung rescue team cohort	6.2 (5.9–6.5)		6.2 (5.9–6.5)		6.2 (5.9–6.5)		6.2 (5.9–6.6)	
DP^a^, cmH_2_O, mean (CI 95%)	Standard protocol-based cohort	13 (12.1–14.1)	*P* = 0.94	13 (12.2–14.8)	*P* < 0.001	13 (11.9–15.2)	*P* < 0.001	13 (11.8–15.1)	*P* < 0.001
	Lung rescue team cohort	13 (12.0–14.3)		10 (8.7–10.4)		9 (8.2–9.9)		8 (7.3–9.6)	
*C*_RS_^b^, mL/cmH_2_O, mean (CI 95%)	Standard protocol-based cohort	35 (31–38)	*P* = 0.41	33 (27–39)	*P* < 0.001	36 (27*–*46)	*P* = 0.003	33 (27–40)	*P* = 0.002
	Lung rescue team cohort	33 (29.7–37.1)		45 (41–49)		48 (41–56)		52 (42–62)	
P_a_/F_i_O_2_, mmHg, mean (CI 95%)	Standard protocol-based cohort	197 (177–217)	*P* = 0.003	224 (203–245)	*P* = 0.001	220 (199–242)	*P* = 0.004	218 (194–242)	*P* = 0.004
	Lung rescue team cohort	154 (127–179)		282 (252–312)		284 (256–312)		276 (243–309)	
RIV No. (%)	Standard protocol-based cohort	49/70 (70)	*P* = 0.47	51/70 (73)	*P* = 0.30	41/70 (58)	*P* = 0.04	39/70 (56)	*P* = 0.005
	Lung rescue team cohort	38/50 (76)		32/50 (64)		20/50 (40)		15/50 (30)	
VIS , mean (CI 95%)	Standard protocol-based cohort	16 (11*–*21)	*P* = 0.79	15 (10–20)	*P* = 0.14	14 (9–20)	*P* = 0.004	15 (6–24)	*P* = 0.001
	Lung rescue team cohort	15 (9–21)		9 (5–12)		5 (2–8)		4 (1–8)	

*Abbreviations*: *PEEP* positive end-expiratory pressure, *CI* confidence interval, *TV* tidal volume, *IBW* ideal body weight, *DP* driving pressure, *CRS* compliance of respiratory system, *RIV* requirement for inotropics and vasopressors, *VIS* vasoactive-inotropic score

^a^Driving pressure is difference between plateau pressure (measured at the end of an end-inspiratory pause) and total positive end-expiratory pressure (measured at the end of an end-expiratory pause)

^b^Respiratory system compliance is the ratio of tidal volume to driving pressure

Information at days 3 and 4 were available for more than 80% of patients and statistics were performed only on available data

Patients in the standard protocol-based cohort were all ventilated according to the ARDSnet lower PEEP/higher F_i_O_2_ table [11] for > 48 h (average 198 ± 278 h). PEEP levels and respiratory mechanics did not change during the first 4 days of ventilation. By day 4, only 10 patients (14%) had improved oxygenation to > 300 mmHg (Table 2 and Additional file 1: Table S1).

Upon admission to the ICU, patients in the lung rescue group were also initially ventilated according to the ARDSnet lower PEEP/higher F_i_O_2_ table [11]. Within 24 h after initiation of mechanical ventilation, the lung rescue team performed esophageal manometry, LRM, and a decremental PEEP trial. As a result of the lung rescue approach, PEEP increased an average of 10 cmH_2_O (9 ± 2 cmH_2_O on day 1 versus 19 ± 4 cmH_2_O on day 2, *P* < 0.001) and end-expiratory transpulmonary pressure passed from − 6.3 ± 3.7 cmH_2_O to + 1.7 ± 3.2 cmH_2_O (*P* < 0.001). All patients in the lung rescue team cohort were ventilated for > 48 h (299 ± 322 h). Comparison of time of ventilation, measured as ventilation-free days, did not reveal a difference between the two cohorts (Table 3).

**Table 3:** Mortality, cause of death, and in-hospital outcomes

	Standard protocol-based cohort (*N* = 70)	Lung rescue team cohort (*N* = 50)	*P*^a^	Hazard ratio (CI 95%)^a^
ICU mortality, *n* (%)	24/70 (34)	9/50 (18)	0.004	0.29 (0.12–0.67)
Hospital mortality, *n* (%)	29/70 (41)	9/50 (18)	< 0.001	0.22 (0.10–0.51)
28-day mortality, *n* (%)	22/70 (31)	8/50 (16)	0.001	0.31 (0.13–0.78)
3-month mortality, *n* (%)	29/70 (41)	11/50 (22)	0.006	0.35 (0.16–0.74)
6-month mortality, *n* (%)	29/70 (41)	11/50 (22)	0.006	0.35 (0.16–0.74)
1-year mortality, *n* (%)	29/70 (41)	11/50 (22)	0.006	0.35 (0.16–0.74)
Cause of death				
Multi-organ failure	27/29	7/11		
Brain injury/advanced cancer	2/29	4/11		
ICU length of stay, days , mean (CI 95%)	13 (9–16)	17 (14–20)	< 0.001	
Days not in ICU at day 28, days, mean (CI 95%)^b^	12 (9–14)	11 (8–13)	0.413	
Hospital length of stay, days, mean (CI 95%)	19 (15–23)	28 (23–33)	< 0.001	
Days not in hospital at day 28, days, mean (CI 95%)^b^	7 (5–9)	5 (3–7)	0.121	
Ventilation-free days, days, mean (CI 95%)^b^	14 (11–16)	15 (12–18)	0.859	
Reintubation, *n* (%)	12/70 (17)	8/50 (16)	0.868	
Tracheostomy, *n* (%)	11/70 (16)	14/50 (28)	0.061	
AKI, *n* (%)	37/70 (52)	26/50 (54)	0.902	
RRT, *n* (%)	16/70 (23)	12/50 (24)	0.884	

*Abbreviations*: *CI* confidence interval, *ICU* intensive care unit, *AKI* acute kidney injury, *RRT* renal replacement therapy

^a^*P* values and hazard ratios for mortality calculated after correction for common ICU confounding factors (APACHE, age, BMI, P_a_O_2_/F_i_O_2_)

^b^If in-hospital death occurred before day 29, the ventilation-free days, the days not in ICU at day 28, and the days not in hospital at day 28 were considered to be zero

In contrast to the standard protocol-based cohort, the lung rescue team cohort showed a remarkable improvement in respiratory mechanics and oxygenation throughout the first 4 days of ventilation. Driving pressure decreased an average of 3.4 cmH_2_O, while compliance of the respiratory system improved an average of 12 mL/cmH_2_O, suggesting considerable lung recruitment. This result was also documented by improved PaO_2_/FiO_2_ ratio from 153 ± 88 mmHg at admission to 282 ± 102 mmHg on day 2, after titration of PEEP (Table 2). On day 4, 28 patients (56%) improved oxygenation to > 300 mmHg.

### Hemodynamics

A large proportion of patients in both groups (70% of patients in the standard protocol-based cohort and 76% of patients in lung rescue team cohort) presented in shock, requiring similar intravenous doses of inotropic and vasopressor agents (16, CI95% 11–21 in the standard protocol-based cohort versus 15, CI95% 9–21 in the lung rescue team cohort, *P* = 0.79). In the first 4 days of ICU admission, the average dose of required inotropics and vasopressors in the standard protocol-based cohort did not substantially change (Table 2). In the lung rescue team cohort, despite the PEEP increase of 10 cmH_2_O, average VIS decreased during the first 4 days of ICU admission. By day 4, the proportion of patients requiring vasopressors decreased to 30%, requiring lower doses of intravenous inotropic agents and vasopressors (Table 2).

To monitor right heart function of hemodynamically unstable patients, the lung rescue team performed bedside transthoracic echocardiography (TTE) before and after titration of ventilation. Both tricuspid annular plane systolic excursion (TAPSE) and peak systolic velocity (S′) were unchanged by LRM and PEEP titration (TAPSE measured in 27 patients 2.3 cm [CI95% 2.10–2.43] before versus 2.2 cm [CI95% 2.05–2.34] after PEEP setting, *P* = 0.51; S′: 15 cm/s [CI95% 13.22–16.88] before versus 14 cm/s [CI95% 12.29–15.84] after PEEP setting, *P* = 0.40), suggesting no adverse impact on right heart function.

### Mortality and in-hospital outcomes

**Fig. 2:**
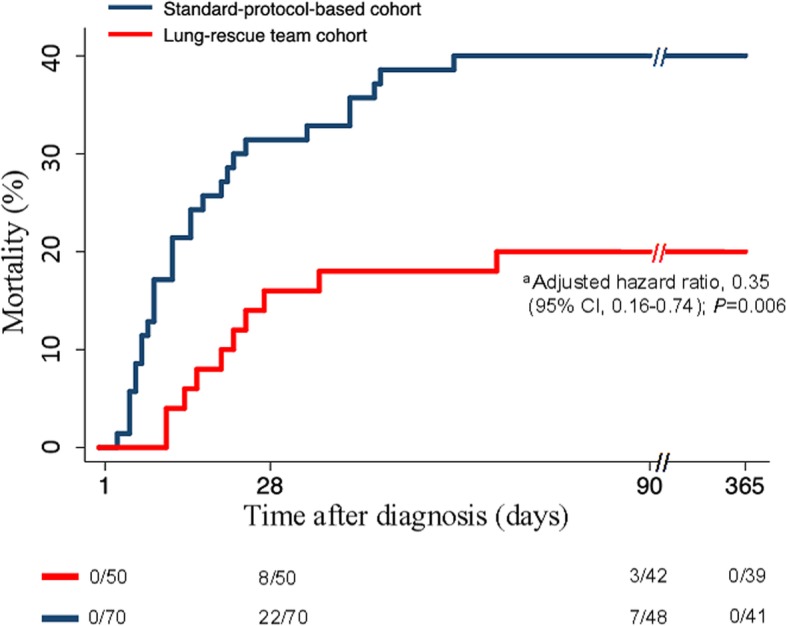
Kaplan-Meier survival of ARDS patients. Survival of patients in the standard protocol-based and lung rescue team cohorts. ^a^Hazard ratio and *P* value calculated after correction for common ICU confounders (APACHE, age, BMI, P_a_O_2_/F_i_O_2_ ratio)

Patients in the standard protocol-based cohort compared to those in the lung rescue team had almost double the risk of dying from an ARDS diagnosis at 28 days and 3 months (Fig. 2 and Table 3).

No deaths occurred in either group after 3 months, and the increased risk of mortality persisted in the standard protocol-based cohort at 6 months and at 1 year after admission. The mortality difference between cohorts was even greater when corrected for potential confounders (BMI, APACHE, age, PaO_2_/FiO_2_ ratio), suggesting that a lung rescue approach is a strong independent variable of improved survival (Fig. 2 and Table 3).

The main cause of death was multi-organ failure in the standard protocol-based cohort (93%; Additional file 1: Table S2) and lung rescue team cohort (64%; Additional file 1: Table S3). In the remaining cases, care was withdrawn in the standard protocol-based cohort for severe and diffuse ischemic brain injury (two patients, 7%) and in the lung rescue team cohort for advanced metastatic cancer (four patients, 36%). All treatments were withdrawn based on patient’s and proxy’s wishes, with the exception of palliative and comfort care.

To evaluate the effects of time on mortality of patients with obesity and ARDS, the two cohorts of patients were subdivided in two further subgroups of equal number of patients. The standard protocol-based cohort mortality was 40% in the first 35 patients (2012–2013) and 42% in the second 35 patients (2013–2014) indicating that mortality did not change over the 3 years. The mortality in the first 25 patients of the lung rescue team cohort was 28% (2015–2016) and 16% in the remaining 25 patients (2016–2017), showing a decrease compared to the standard protocol-based cohort.

When mortality was accounted for, days not in hospital at day 28 [25] and days not in ICU at day 28 [25] were similar in the two groups. The increased ICU length of stay and hospital length of stay in the lung rescue team cohort have to be ascribed exclusively to improved survival (Table 3). No differences were observed in the incidence of reintubation, tracheostomy, and renal acute injury between cohorts.

### Safety of procedures

No safety concerns were recorded associated with the lung rescue procedures (please see Additional file 1: Supplementary Materials).

## Discussion

### Major findings

The implementation of a lung rescue team to individually titrate mechanical ventilation according to physiological parameters in patients with class III obesity and ARDS was associated with significantly decreased mortality at 28 days and at 3 months compared with use of the ARDSnet lower PEEP/higher F_i_O_2_ table. The mortality difference persisted at 1-year follow-up.

The largest epidemiologic study on ARDS was conducted in 2016 and reported an overall ICU mortality of 35.3% (95%CI 33.3%–37.2%) and hospital mortality of 40.0% (95CI 38.1%–42.1%) [7], similar to what we observed in the standard protocol-based cohort treated according to the ARDSnet protocol. In 2019, two large randomized US trials in ARDS reported a mortality rate at 1 year of 44% [26] and at 90 days of 42% [25]. MGH was part of those two studies. Thus, the improved survival observed in the lung rescue group might be attributed to a prompt and sustained improvement in cardiopulmonary physiology following the individualized titration of mechanical ventilation.

In the USA, the largest trial in ARDS patients that systematically changed the common practice of mechanical ventilation was the original ARDSnet trial [9], a study sponsored by NIH and completed in 2000. This trial developed simple and clear mechanical ventilation protocols (ARDSnet tables) to guide clinicians to deliver mechanical ventilation for ARDS patients [11]. However, obesity was a criterion of exclusion from the trial and for many subsequent ARDS trials focused on best practice of mechanical ventilation [9, 11, 14, 15, 27].

Despite the high prevalence of class III obesity [2] in the USA and increasing health issues related to this condition, to date no study has primarily evaluated this population with ARDS. The PROBESE study [28] showed no difference between two protocolized ventilator strategies (low PEEP, [4 cmH_2_O] versus lung recruitment maneuvers and high PEEP [12 cmH_2_O]) during general anesthesia for surgery in patients with obesity and without ARDS. To our knowledge, our observational study is the first to investigate the effects on survival of an alternative individualized and physiologically driven approach of care, rather than use of ARDSnet protocols in patients with class III obesity and ARDS. In the absence of definitive guidance, over the past years we have meticulously studied pulmonary physiology and hemodynamics in mechanically ventilated patients with obesity [17–19].

The question in ARDS patients is whether non-functional atelectatic lung can be re-opened without subjecting the normal lung to further injury.

ARDS patients are often said to have a “baby lung,” a reduced lung volume but with a highly variable amount of recruitable lung parenchyma [29]. The increased pleural pressure in patients with class III obesity [16–19] causes atelectasis of > 40% of lung parenchyma. Atelectasis in patients with class III obesity can easily be recruited [17, 19]. Our study intervention sought to maximize recruitment of atelectatic lung. This occurred, as shown by the decrease in driving pressure in the lung rescue group. Amato et al. [12] documented that low driving pressure predicts increased survival in ARDS patients. Driving pressure declined only in patients who received individualized physiologic measurements in the second cohort. Decreased driving pressure, improved compliance of the respiratory system, and improved oxygenation all confirm lung recruitment [30].

In both the cohorts of patients, pressure-volume curves were not performed, thus careful airway closure was not estimated; however, as shown by Grieco [31] in people with obesity, airway closure might be a common phenomenon in the obese population and could co-exist with alveolar derecruitment in our patients as well. Despite the real value of alveolar pressure, it is unknown when airway closure is detected, it was shown [31] that theoretically it could be close to the airway opening pressure; consequently, the alveolar pressure at the end of expiration is independent of the applied PEEP when its value is below the opening airway pressure.

Contrary to the common association between high levels of airway pressure and reduced right heart function with hemodynamic impairment, the lung rescue approach was associated with a decreased proportion of patients requiring vasoactive and inotropic agents. During LRM and after titration of PEEP, most patients’ hemodynamics remained unchanged, as shown by TTE right systolic measurements and unchanged doses of vasoactive and inotropic drug infusions. In contrast to the recently published ART trial [13], a large study of ARDS patients, we found neither barotrauma nor cardiac arrest in our population. Differences in response to increased airway pressure found in the ART trial compared to hemodynamic stability observed in the lung rescue cohort might be explained by the large amount of recruitable lung parenchyma. When atelectatic lung is recruited, there is a decrease in pulmonary vascular resistance and right heart workload. While our study did not invasively measure cardiac output, pulmonary pressures, or filling pressures, our prior work using a porcine model of obesity documented unchanged pulmonary vascular resistance and hemodynamics with both LRM and decremental PEEP trial [18]. In patients treated according to the lung rescue approach, hemodynamic stability continues if ventilation can establish a homogeneous distribution of ventilation, physiological lung volumes with low transpulmonary pressures, and minimal alveolar overstretch, even when higher levels of airway pressures are required. Prior physiological studies have confirmed that hemodynamics of critically ill patients with high pleural pressure and obesity tolerate LRM and increased airway pressures [17, 18].

### Limitations

First, this report is not a randomized controlled trial but a single-center retrospective study with a limited number of patients, evaluating two cohorts of patients treated with different approaches to mechanical ventilation. Notably, at ICU admission, patients in the lung rescue team cohort had worse oxygenation, which is associated with a higher severity of illness, than patients in the standard protocol-based cohort [32]. Despite increased critical illness, the lung rescue team cohort had decreased mortality in multivariate analysis after adjusting for common ICU mortality confounders, including age, BMI, APACHE, and PaO_2_/FiO_2_ ratio (Additional file 1: Table S4). The strength of the physiological rationale and improvement in mortality suggests future multicenter prospective randomized trials should be done to confirm these findings.

Second, survival benefits observed in the lung rescue team cohort might result from recent improvements in care of patients with obesity and novel ARDS therapies. However, since 2012 at MGH, there have not been changes in the care of patients with class III obesity, in titration of mechanical ventilation, or in treatment of septic shock, except those discussed in this study. Further, as mentioned in the “Methods”, none of our patients were enrolled in any MGH clinical trials. Although we cannot exclude other factors beyond our knowledge that might have affected the outcomes in the two cohorts, we know that, accounting for patients enrolled in trials, ARDS mortality did not change at MGH over the past 10 years (unpublished data) and did not change in the most recent US ARDS trials [25, 26].

Third, benefits associated with the lung rescue approach might be difficult to reproduce in other centers, unless a dedicated team has expertise in advanced measurements of lung physiology and hemodynamics. In 2014, we implemented a lung rescue team at MGH to optimize mechanical ventilation in patients with class III obesity. The research fellows that participated in the specialized team received ongoing training over a year in measurements of respiratory and cardiac physiology, including use of TTE, transpulmonary pressure measurement, and respiratory mechanics, which allowed personalized assessment of each patient in the lung rescue team cohort.

Fourth, despite we considered patients with an average BMI higher than 50 kg/m^2^, in the present study, we did not take into account the possible differences between abdominal and non-abdominal obesity and their correlation with BMI.

## Conclusions

To our knowledge, this is the first observational study to specifically investigate the impact of different mechanical ventilation approaches in patients with class III obesity and ARDS on survival. We found that in patients with an average BMI of > 50 kg/m^2^, an individualized lung rescue approach based on individualized cardiopulmonary physiology is associated with a decreased in-hospital mortality. Based on our findings and considering the increasingly large group of hypoxic ARDS patients with obesity, the present study justifies the conduction of a randomized control trial testing whether a titration of mechanical ventilation based on an individualized strategy with a dedicated health professionals’ team might be superior to a fixed protocol based on the ARDSnet lower PEEP/higher F_i_O_2_ table.

## Supplementary information

Additional file 1: Supplementary material. Additional information and tables about methods and results.

## Abbreviations

BMI: Body mass index
ICU: Intensive care unit
ARDS: Acute respiratory distress syndrome
PEEP: Positive end-expiratory pressure
LRM: Lung recruitment maneuver
TTE: Transthoracic echocardiography
TAPSE: Tricuspid annular plane systolic excursion
S′: Peak systolic velocity
