# Supplementary material for: A lung rescue team improves survival in obesity with acute respiratory distress syndrome
Source: Crit Care. 2020 Jan 15;24:4. doi: 10.1186/s13054-019-2709-x (PMC6961369; doi:10.1186/s13054-019-2709-x)
Supplement: Supplementary file 1 — Supplementary material. Additional information and tables about methods and results. [file 13054_2019_2709_MOESM1_ESM.docx]

**A lung rescue team improves survival in obesity with acute respiratory distress syndrome**

Florio G, et al. For the investigators of the lung rescue team.

**Supplement materials**

**METHODS**

- **Screening of patients**
- **Data source**
- **Recording and measurements**
- **Interventions**

**RESULTS**

- **Safety of lung rescue procedures**
- **Table S1**. Ventilation settings and hemodynamics – standard protocol-based cohort and lung rescue team cohort
- **Table S2**. Time of death and causes of death – standard protocol-based cohort
- **Table S3**. Time of death and causes of death – lung rescue team cohort
- **Table S4**. Adjusted and unadjusted mortality

**REFERENCES**

**METHODS**

Screening of patients

The Respiratory Care Department monitored and tracked all ventilated hospital patients. Patient data, including mechanical ventilation data, were recorded by the intensive care unit (ICU) respiratory therapist for both cohorts. Since 2012, all patients with class III obesity requiring mechanical ventilation were screened 24 hours/day and 7 days/week for diagnosis of acute respiratory distress syndrome (ARDS)^1^ by the respiratory therapist who alerted the lung rescue team.

Data sources

Data were collected from medical records of the patient’s hospital stay, including charting of physicians, nurses, and respiratory therapists. Survival data at one year were determined by accessing the last updated notes and follow-up visits on MGH medical charts. In the absence of medical notes, patients were contacted by phone.

Recordings and measurements

During ICU admission, the following patient characteristics were collected: sex, race, age, body mass index (BMI), cause of hospital admission (elective surgery, urgent surgery, medical), severity of illness (APACHE II, SOFA), and comorbidities.

During the first four days of mechanical ventilation, respiratory mechanics, gas exchange, and hemodynamics were recorded, specifically: tidal volume, respiratory rate, total positive end-expiratory pressure (PEEP) (measured after an end-expiratory pause), plateau pressure (measured after an end-inspiratory pause), driving pressure (defined as difference between plateau pressure and total PEEP), intrinsic PEEP (defined as difference between total and set PEEP), compliance of respiratory system (defined as ratio between expiratory tidal volume and driving pressure), pH, P_a_O_2_, P_a_CO_2_, blood arterial pressure, heart rate, and central venous pressure. Vasoactive-inotropic score (VIS), introduced by Wernowsky^2^ and modified by Davidson,^3^ was used to summarize the levels of vasoactive-inotropic agents. VIS was calculated using the following formula:

**VIS** = dopamine dose (mcg/kg/min) + dobutamine dose (mcg/kg/min) + 100 × epinephrine dose (mcg/kg/min) + 10 × milrinone dose (mcg/kg/min) + 10,000 × vasopressin dose (units/kg/min) + 100 × norepinephrine dose (mcg/kg/min) + 10 × phenylephrine dose (mcg/kg/min)

The weight used to calculate VIS was 130% of the ideal body weight (IBW).

The following outcomes were recorded: ICU and hospital length of stay, ventilator-free days^4^, days not in ICU at day 28^5^, days not in hospital at day 28^5^, incidence of reintubation, tracheostomy, acute kidney injury, and need for renal replacement therapy. Survival was assessed at 28 days, three months, six months, and one year.

In the cohort of patients treated by the lung rescue team, a nasogastric tube containing an esophageal balloon (AVEA Ventilator Nasogastric Pressure Monitoring Tube Set, CareFusion, Yorba Linda, CA) was inserted within a 24-hour interval after initiation of mechanical ventilation. Balloon positioning and inflation volume were verified as previously described,^6,7^ Airway and esophageal pressure were recorded during the first four days of mechanical ventilation. Airway and esophageal pressure at the end of inspiration and at the end of exhalation were also obtained. Transpulmonary pressure at the end of exhalation (P_L_E) was calculated as the difference between airway pressure and esophageal pressure (P_ES_), both at the end of exhalation.

Before and after lung recruitment maneuver (LRM) and PEEP setting, right heart trans-thoracic echocardiography (TTE) was performed with measurement of tricuspid annular plane systolic excursion (TAPSE)^8^ and peak systolic velocity (S’).^9^

Interventions (Lung rescue team approach)

1. LRM is a brief increase in airway pressure to recruit atelectatic and collapsed regions of lung and is performed in a stepwise fashion. First, the patient’s ventilatory mode was switched to pressure control ventilation, with PEEP set at 15 cmH_2_O, respiratory rate at 10-20 breaths/minute based on patient ventilation demand, inspiratory/expiratory ratio of 1:1, and pressure control level of 10-15 cmH_2_O based on ventilation requirement. Every 30 seconds, PEEP was increased by 5 cmH2O to obtain a final peak inspiratory pressure of 45-50 cmH_2_O. The total interval for recruitment of the lungs never exceeded 2.5 min. Hemodynamics and respiratory parameters were closely monitored, and the LRM maneuver was immediately interrupted (with re-establishment of baseline ventilation) if any of the following occurred: heart rate (HR) of >130/min or <60/min, persistent systolic blood pressure (SBP) of <90 mmHg, incremental increase in vasopressors (>5 mcg/min for norepinephrine, dopamine, or dobutamine; >2 mcg/min for epinephrine; or >50 mcg/min for phenylephrine), or desaturation (SpO2 decrease of 5%).^10^

2. Esophageal manometry was recorded during the entire respiratory procedure to estimate inspiratory and expiratory transpulmonary pressures.^11^

3. A decremental PEEP trial was performed following the LRM by increasing PEEP to a level 10 cmH_2_O higher than the PEEP level that equaled 0 cmH_2_O transpulmonary pressure and not exceeding a maximum of 30 cmH_2_O PEEP and plateau pressure of 50 cmH_2_O. After 2 min, plateau pressure and total PEEP were measured. Driving pressure was calculated and PEEP was decreased in a stepwise manner by 2 cmH_2_O while monitoring regional ventilation by electrical impedance tomography. PEEP level resulting in the lowest driving pressure, while keeping end-expiratory transpulmonary pressure between -4 cmH_2_O and 4 cmH_2_O in the absence of deterioration of hemodynamics (as specified in point 4 below) and avoiding atelectasis and lung overdistension, identified lung rescue PEEP of each patient.^10^

4. Before LRM and setting PEEP, the clinical team assessed fluid responsiveness and fluid administration. In most hemodynamically unstable patients, evaluation of TAPSE^8^ and S’^9^ by TTE was performed. In the lung rescue team cohort, after optimal PEEP was determined, patients were continuously monitored. If hemodynamics deteriorated within the subsequent 6 hours, PEEP was modified per the ARDSnet PEEP/FiO2 table.^12^ We defined hemodynamic deterioration from ventilation titration using the lung rescue paradigm as any of the following: (I) change of hemodynamics (HR of >130/min or <60/min, SBP persistently <90 mmHg); (II) change of TTE (TAPSE < 1.4 cm and S’ <10 cm/s, or less if pre-PEEP values were lower); or (III) incremental increase in vasopressors (>5 mcg/min for norepinephrine, dobutamine or dopamine; >2 mcg/min for epinephrine; >50 mcg/min for phenylephrine; or >0.2 mcg/min for milrinone)^10^.

**RESULTS**

Safety of lung rescue procedures

In the lung rescue group, all 50 patients tolerated the increase of PEEP. In no case was PEEP decreased due to hemodynamic compromise. No patient required starting new inotropics or vasopressors. Four patients required a temporary increased dose of intravascular infusion of inotropics and vasopressor (VIS increased an average of 4 points in these patients; range: 1–6), of which three had decreased VIS within 24 hours. No pneumothorax was recorded while ventilation was managed according the lung rescue approach.

| **Variable** | **Group** | **Day 1** | | **Day 2** | | **Day 3** | | **Day 4** | |
| --- | --- | --- | --- | --- | --- | --- | --- | --- | --- |
| PEEP, cmH_2_O  (CI 95%) | Standard protocol-based cohort | 9  (8–10) | *P*=0.20 | 9  (8–10) | *P*<0.001 | 9  (8–10) | *P*<0.001 | 9  (8–10) | *P*<0.001 |
|  | Lung rescue  team cohort | 9  (9–10) |  | 19  (18–20) |  | 20  (18–21) |  | 20  (18–21) |  |
| RR, breaths/min (CI 95%) | Standard protocol-based cohort | 23  (21–24) | *P*=0.59 | 24  (22–25) | *P*=0.94 | 22  (20–24) | *P*=0.20 | 22  (20–24) | *P*=0.82 |
|  | Lung rescue  team cohort | 23  (21–24) |  | 24  (22–25) |  | 24  (22–25) |  | 23  (21–24) |  |
| TV, mL  (CI 95%) | Standard protocol-based cohort | 421  (398–445) | *P*=0.74 | 414  (392–436) | *P*=0.83 | 423  (399–447) | *P*=0.89 | 426  (402–449) | *P*=0.63 |
|  | Lung rescue  team cohort | 414  (392–436) |  | 411  (390–431) |  | 417  (393–440) |  | 420  (390–450) |  |
| TV/IBW, mL/kg  (CI 95%) | Standard protocol-based cohort | 6.4  (6.2–6.6) | *P*=0.33 | 6.5  (6.3–6.8) | *P*=0.10 | 6.4  (6.1–6.6) | *P*=0.21 | 6.5  (6.3–6.7) | *P*=0.11 |
|  | Lung rescue  team cohort | 6.2  (5.9–6.5) |  | 6.2  (5.9–6.5) |  | 6.2  (5.9–6.5) |  | 6.2  (5.9–6.6) |  |
| P_plat_,  cmH_2_O  (CI 95%) | Standard protocol-based cohort | 23  (21–24) | *P*=0.21 | 25  (23–27) | *P*<0.001 | 24  (22–26) | *P*<0.001 | 25  (23–27) | *P*=0.049 |
|  | Lung rescue  team cohort | 24  (22–25) |  | 29  (28–30) |  | 29  (28–31) |  | 28  (26–30) |  |
| DP, cmH_2_O  (CI 95%)^a^ | Standard protocol-based cohort | 13  (12.1–14.1) | *P*=0.94 | 13  (12.2–14.8) | *P*<0.001 | 13  (11.9–15.2) | *P*<0.001 | 13  (11.8–15.1) | *P*<0.001 |
|  | Lung rescue  team cohort | 13  (12.0–14.3) |  | 10  (8.7–10.4) |  | 9  (8.2–9.9) |  | 8  (7.3–9.6) |  |
| PEEP_i_, cmH_2_O  (CI 95%) | Standard protocol-based cohort | 0.7  (0.5–0.9) | *P*=0.11 | 0.9  (0.6–1.2) | *P*=0.08 | 0.5  (0.2–0.9) | *P*=0.36 | 0.6  (0.3–0.9) | *P*=0.23 |
|  | Lung rescue  team cohort | 0.5  (0.3–0.6) |  | 0.6  (0.4–0.8) |  | 0.4  (0.2–0.6) |  | 0.4  (0.1–0.6) |  |
| C_RS_, mL/cmH_2_O  (CI 95%)^b^ | Standard protocol-based cohort | 35  (31–38) | *P*=0.41 | 33  (27–39) | *P*<0.001 | 36  (27–46) | *P*=0.003 | 33  (27–40) | *P*=0.002 |
|  | Lung rescue  team cohort | 33  (29.7–37.1) |  | 45  (41–49) |  | 48  (41–56) |  | 52  (42–62) |  |
| pH  (CI 95%) | Standard protocol-based cohort | 7.33  (7.31–7.36) | *P*=0.24 | 7.35  (7.33–7.37) | *P*=0.11 | 7.37  (7.35–7.38) | *P*=0.10 | 7.38  (7.35–7.40) | *P*=0.08 |
|  | Lung rescue  team cohort | 7.31  (7.29–7.34) |  | 7.34  (7.31–7.36) |  | 7.35  (7.33–7.37) |  | 7.36  (7.33–7.38) |  |
| P_a_/F_i_O_2_, mmHg  (CI 95%) | Standard protocol-based cohort | 197  (177–217) | *P*=0.003 | 224  (203–245) | *P*=0.001 | 220  (199–242) | *P*<0.001 | 218  (194–242) | *P*=0.004 |
|  | Lung rescue  team cohort | 154  (127–179) |  | 282  (252–312) |  | 284  (256–312) |  | 276  (243–309) |  |
| P_a_CO_2_, mmHg  (CI 95%) | Standard protocol-based cohort | 47  (43–50) | *P*=0.11 | 44  (42–46) | *P*=0.06 | 44  (41–47) | *P*=0.78 | 46  (43–49) | *P*=0.46 |
|  | Lung rescue  team cohort | 49  (45–51) |  | 47  (45–50) |  | 44  (41–48) |  | 45  (41–49) |  |
| SBP,  mmHg  (CI 95%) | Standard protocol-based cohort | 117  (112–122) | *P*=0.17 | 116  (110–121) | *P*=0.06 | 115  (109–121) | *P*=0.45 | 117  (110–123) | *P*=0.43 |
|  | Lung rescue  team cohort | 120  (115–124) |  | 120  (116–124) |  | 118  (115–123) |  | 119  (115–123) |  |
| DBP, mmHg  (CI 95%) | Standard protocol-based cohort | 6  (58–64) | *P*=0.70 | 58  (56–60) | *P*=0.47 | 57  (55–60) | *P*=0.57 | 57  (54–59) | *P*=0.21 |
|  | Lung rescue  team cohort | 60  (57–63) |  | 57  (55–60) |  | 59  (56–62) |  | 59  (56–63) |  |
| MBP, mmHg  (CI 95%) | Standard protocol-based cohort | 80  (77–83) | *P*=0.72 | 77  (75–80) | *P*=0.38 | 77  (74–80) | *P*=0.37 | 76  (73–79) | *P*=0.26 |
|  | Lung rescue  team cohort | 80  (77–82) |  | 78  (76–81) |  | 78  (75–81) |  | 79  (76–82) |  |
| HR, beats/min  (CI 95%) | Standard protocol-based cohort | 87  (82–91) | *P*=0.98 | 84  (80–89) | *P*=0.73 | 83  (79–87) | *P*=0.62 | 85  (80–89) | *P*=0.42 |
|  | Lung rescue  team cohort | 88  (82–93) |  | 85  (80–89) |  | 83  (78–89) |  | 82  (78–86) |  |
| CVP, mmHg  (CI 95%) | Standard protocol-based cohort | 15  (13–16) | *P*=0.80 | 14  (13–16) | *P*=0.61 | 13  (12–15) | *P*=0.29 | 13  (12–14) | *P*=0.09 |
|  | Lung rescue  team cohort | 14  (13-16) |  | 15  (14–16) |  | 14  (13–15) |  | 14  (13–15) |  |
| RIV, n (%) | Standard protocol-based cohort | 49/70 (70) | *P*=0.47 | 51/70 (33) | *P*=0.30 | 41/70 (58) | *P*=0.04 | 39/70 (56) | *P*=0.005 |
|  | Lung rescue  team cohort | 38/50 (76) |  | 32/50 (64) |  | 20/50 (40) |  | 15/50 (30) |  |
| VIS  (CI 95%) | Standard protocol-based cohort | 16  (11–21) | *P*=0.79 | 15  (10–20) | *P*=0.14 | 14  (9–20) | *P*=0.004 | 15  (6–24) | *P*=0.001 |
|  | Lung rescue  team cohort | 15  (9–21) |  | 9  (5–12) |  | 5  (2–8) |  | 4  (1–8) |  |

**Table 1s. Ventilation settings and hemodynamic – standard protocol-based cohort and lung rescue team cohort**

**Table S1. Ventilation settings and hemodynamics – Standard protocol-based cohort and lung rescue team cohort**

Abbreviations: PEEP, positive end-expiratory pressure; CI, confidence interval; RR, respiratory rate; TV, tidal volume; IBW, ideal body weight; P_plat_, plateau pressure; DP, driving pressure; PEEP_i_, intrinsic positive end-expiratory pressure; C_RS_, respiratory system compliance; SBP, systolic blood pressure; DBP, diastolic blood pressure; MBP, mean blood pressure; HR, heart rate; CVP, central venous pressure; RIV, requirement for inotropics and vasopressors; VIS, vasoactive-inotropic score

^a^ Driving pressure is difference between plateau pressure (measured at the end of an end-inspiratory pause) and total positive end-expiratory pressure (measured at the end of an end-expiratory pause).

^b^ Respiratory system compliance is ratio of tidal volume to driving pressure.

Information at day 3 and 4 were available for more than 80% of patients and statistics were performed only on available data.

| **Number of deaths** | **Number of survived days after intubation** | **Cause of death** | |
| --- | --- | --- | --- |
|  |  | **Multi-organ failure** | **Others** |
| 1 | 20 | Heart, lung |  |
| 2 | 12 | Heart, lung, kidney |  |
| 3 | 5 |  | Post anoxic coma |
| 4 | 24 | Heart, lung, kidney |  |
| 5 | 17 | Heart, lung, kidney |  |
| 6 | 9 | Heart, lung, kidney |  |
| 7 | 17 |  | Intracerebral hemorrhage |
| 8 | 7 | Heart, lung |  |
| 9 | 9 | Heart, lung |  |
| 10 | 3 | Heart, lung, kidney |  |
| 11 | 12 | Heart, lung, kidney |  |
| 12 | 12 | Heart, lung, kidney |  |
| 13 | 41 | Heart, lung, kidney |  |
| 14 | 8 | Heart, lung |  |
| 15 | 6 | Heart, lung, kidney |  |
| 16 | 6 | Heart, lung, kidney |  |
| 17 | 7 | Heart, lung |  |
| 18 | 5 | Heart, lung, kidney |  |
| 19 | 15 | Heart, lung |  |
| 20 | 34 | Heart, lung |  |
| 21 | 22 | Heart, lung, kidney |  |
| 22 | 9 | Heart, lung |  |
| 23 | 5 | Heart, lung |  |
| 24 | 41 | Heart, lung, kidney |  |
| 25 | 5 | Heart, lung, kidney |  |
| 26 | 21 | Heart, lung |  |
| 27 | 15 | Heart, lung |  |
| 28 | 45 | Heart, lung |  |
| 29 | 58 | Heart, lung |  |

**Table 2s. Time of death and cause of death – standard-protocol based cohort**

| **Number of deaths** | **Number of survived days after intubation** | **Cause of death** | |
| --- | --- | --- | --- |
|  |  | **Multi-organ failure** | **Others** |
| 1 | 11 | Heart, lung, kidney |  |
| 2 | 27 | Heart, lung, kidney |  |
| 3 | 16 |  | Advanced cancer |
| 4 | 29 | Heart, lung |  |
| 5 | 11 | Heart, lung |  |
| 6 | 20 | Heart, lung, kidney |  |
| 7 | 65 | Heart, lung |  |
| 8 | 14 | Heart, lung, kidney |  |
| 9 | 24 |  | Advanced cancer |
| 10 | 39 |  | Advanced cancer |
| 11 | 22 |  | Advanced cancer |

**Table S3. Time of death and causes of death – lung rescue team cohort**

|  | **Standard protocol-based cohort**  **(N = 70)** | **Lung rescue team cohort**  **(N = 50)** | **Unadjusted**  ***P*** | **Unadjusted hazard ratio**  **(CI 95%)** | ***^a^*Adjusted**  ***P*** | **^a^Adjusted hazard ratio**  **(CI 95%)** |
| --- | --- | --- | --- | --- | --- | --- |
| ICU mortality, n (%) | 24/70 (34) | 9/50 (18) | 0.005 | 0.33 (0.15–0.72) | 0.004 | 0.29 (0.12–0.67) |
| Hospital mortality, n (%) | 29/70 (41) | 9/50 (18) | 0.001 | 0.28 (0.13–0.61) | <0.001 | 0.22 (0.10–0.51) |
| 28-day mortality, n (%) | 22/70 (31) | 8/50 (16) | 0.046 | 0.43 (0.19–0.98) | 0.012 | 0.32 (0.13–0.78) |
| 3-month mortality, n (%) | 29/70 (41) | 11/50 (22) | 0.023 | 0.44 (0.22–0.89) | 0.006 | 0.35 (0.16–0.74) |
| 6-month mortality, n (%) | 29/70 (41) | 11/50 (22) | 0.023 | 0.44 (0.22–0.89) | 0.006 | 0.35 (0.16–0.74) |
| 1-year mortality, n (%) | 29/70 (41) | 11/50 (22) | 0.023 | 0.44 (0.22–0.89) | 0.006 | 0.35 (0.16–0.74) |

**Table S4. Adjusted and unadjusted mortality.**

Abbreviations: ICU, intensive care unit

^a^ *P*-values and hazard ratios for mortality calculated after correction for common ICU confounding factors (APACHE, age, BMI, P_a_O_2_/F_i_O_2_).

**REFERENCES**

1. ARDS Definition Task Force, Ranieri VM, Rubenfeld GD, Thompson BT, Ferguson ND, Caldwell E, Fan E, Camporota L, Slutsky AS: Acute respiratory distress syndrome: the Berlin Definition. JAMA 2012; 307:2526–33

2. Wernovsky G, Wypij D, Jonas RA, Mayer JE, Hanley FL, Hickey PR, Walsh AZ, Chang AC, Castañeda AR, Newburger JW, Wessel DL: Postoperative course and hemodynamic profile after the arterial switch operation in neonates and infants. A comparison of low-flow cardiopulmonary bypass and circulatory arrest. Circulation 1995; 92:2226–35

3. Davidson J, Tong S, Hancock H, Hauck A, Cruz E da, Kaufman J: Prospective validation of the vasoactive-inotropic score and correlation to short-term outcomes in neonates and infants after cardiothoracic surgery. Intensive Care Med 2012; 38:1184–90

4. Acute Respiratory Distress Syndrome Network, Brower RG, Matthay MA, Morris A, Schoenfeld D, Thompson BT, Wheeler A: Ventilation with lower tidal volumes as compared with traditional tidal volumes for acute lung injury and the acute respiratory distress syndrome. N Engl J Med 2000; 342:1301–8

5. National Heart, Lung and BIPCTN, Moss M, Huang DT, Brower RG, Ferguson ND, Ginde AA, Gong MN, Grissom CK, Gundel S, Hayden D, Hite RD, Hou PC, Hough CL, Iwashyna TJ, Khan A, Liu KD, Talmor D, Thompson BT, Ulysse CA, Yealy DM, Angus DC: Early Neuromuscular Blockade in the Acute Respiratory Distress Syndrome. N Engl J Med 2019; 380:1997–2008

6. Mojoli F, Iotti GA, Torriglia F, Pozzi M, Volta CA, Bianzina S, Braschi A, Brochard L: In vivo calibration of esophageal pressure in the mechanically ventilated patient makes measurements reliable. Crit Care 2016; 20:98

7. Baydur A, Behrakis PK, Zin WA, Jaeger M, Milic-Emili J: A simple method for assessing the validity of the esophageal balloon technique. Am Rev Respir Dis 1982; 126:788–91

8. Kaul S, Tei C, Hopkins JM, Shah PM: Assessment of right ventricular function using two-dimensional echocardiography. Am Heart J 1984; 107:526–31

9. Meluzín J, Spinarová L, Bakala J, Toman J, Krejcí J, Hude P, Kára T, Soucek M: Pulsed Doppler tissue imaging of the velocity of tricuspid annular systolic motion; a new, rapid, and non-invasive method of evaluating right ventricular systolic function. Eur Heart J 2001; 22:340–8

10. Fumagalli J, Santiago RRS, Teggia Droghi M, Zhang C, Fintelmann FJ, Troschel FM, Morais CCA, Amato MBP, Kacmarek RM, Berra L, Lung Rescue Team Investigators: Lung Recruitment in Obese Patients with Acute Respiratory Distress Syndrome. Anesthesiology 2019; 130:791–803

11. Talmor D, Sarge T, Malhotra A, O’Donnell CR, Ritz R, Lisbon A, Novack V, Loring SH: Mechanical Ventilation Guided by Esophageal Pressure in Acute Lung Injury. N Engl J Med 2008; 359:2095–104

12. Brower RG, Lanken PN, MacIntyre N, Matthay MA, Morris A, Ancukiewicz M, Schoenfeld D, Thompson BT, National Heart, Lung and BIACTN: Higher versus lower positive end-expiratory pressures in patients with the acute respiratory distress syndrome. N Engl J Med 2004; 351:327–36
